# Correction: Biodegradable smart materials with self-healing and shape memory function for wound healing

**DOI:** 10.1039/d5ra90144h

**Published:** 2026-01-05

**Authors:** Siqin Sun, Chaoxian Chen, Jianghong Zhang, Jianshe Hu

**Affiliations:** a Department of Chemistry, College of Science, Northeastern University Shenyang 110819 P. R. China hujs@mail.neu.edu.cn; b Department of Material Science and Engineering, Key Laboratory of Polymer Chemistry and Physics of Ministry of Education, Peking University Beijing 100871 P. R. China Chenchaoxian1001818@pku.edu.cn

## Abstract

Correction for ‘Biodegradable smart materials with self-healing and shape memory function for wound healing’ by Siqin Sun *et al.*, *RSC Adv.*, 2023, **13**, 3155–3163, https://doi.org/10.1039/D2RA07493A.

The authors regret that incorrect panels were included within Fig. 6. The correct Fig. 6 is as shown here.

**Fig. 6:**
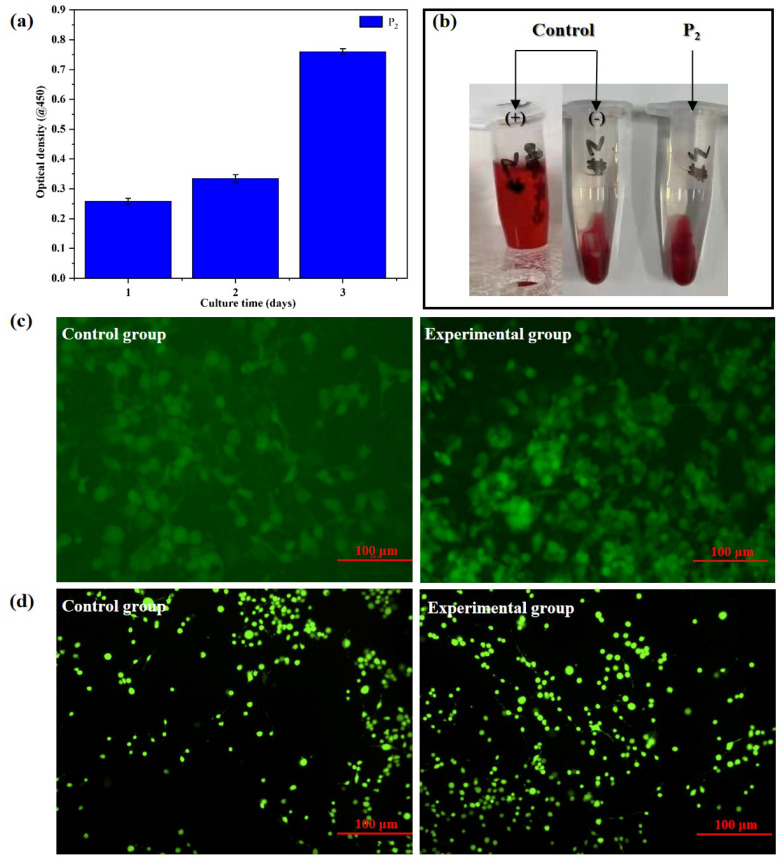
(a) The OD values of the polymer. (b) Results of hemolysis tests of the polymer. (c) Results of confocal tests of the polymer. (d) Clone-forming of HUVECs.

An independent expert has viewed the corrected images and has concluded that they are consistent with the discussions and conclusions presented.

The Royal Society of Chemistry apologises for these errors and any consequent inconvenience to authors and readers.

